# Ten simple rules to make your computing more environmentally sustainable

**DOI:** 10.1371/journal.pcbi.1009324

**Published:** 2021-09-20

**Authors:** Loïc Lannelongue, Jason Grealey, Alex Bateman, Michael Inouye

**Affiliations:** 1 Cambridge Baker Systems Genomics Initiative, Department of Public Health and Primary Care, University of Cambridge, Cambridge, United Kingdom; 2 British Heart Foundation Cardiovascular Epidemiology Unit, Department of Public Health and Primary Care, University of Cambridge, Cambridge, United Kingdom; 3 Health Data Research UK Cambridge, Wellcome Genome Campus and University of Cambridge, Cambridge, United Kingdom; 4 Cambridge Baker Systems Genomics Initiative, Baker Heart and Diabetes Institute, Melbourne, Victoria, Australia; 5 Department of Mathematics and Statistics, La Trobe University, Melbourne, Australia; 6 European Molecular Biology Laboratory, European Bioinformatics Institute (EMBL-EBI), Wellcome Genome Campus, Hinxton, United Kingdom; 7 British Heart Foundation Centre of Research Excellence, University of Cambridge, Cambridge, United Kingdom; 8 The Alan Turing Institute, London, United Kingdom; Carnegie Mellon University, UNITED STATES

## Introduction

We are in the midst of a man-made climate emergency, and yet, there is a widespread underappreciation in our community as to the effects of our computations on carbon emissions and global warming. Global temperatures are rising, largely caused by greenhouse gas (GHG) emissions, which is leading to melting of the ice caps and permafrost. Acidification of the oceans is threatening the entire ocean ecosystem. Global biodiversity is rapidly declining across the globe. Although science can contribute to our understanding of the environment and has great potential to develop strategies and technology to slow down global warming, we do not have a free pass when it comes to the environmental impact of our work. There is little doubt that data science in particular will be a key tool to tackle climate change, but we often forget to consider how our own work also contributes to the problem. The infrastructure we use and the algorithms and code that we write and run all consume large quantities of electricity, whose production is responsible for significant GHG emissions.

It was estimated that the IT sector was responsible for 2% to 6% of global CO_2_ emissions in 2020, a share that could grow to 20% by 2030 [1]. Data centres themselves have a substantial carbon footprint of around 100 megatons of CO_2_e (comparable to American commercial aviation) emitted just from the yearly generation of 200 TWh of electricity [2]. Projections estimate that this footprint will increase by 2- to 9-fold in the next decade [3], with an electricity usage potentially as high as 974 TWh in 2030 [4]. This doesn’t even include the environmental impact of producing and disposing of the hardware needed for computation. Cryptocurrencies are another source of concerns, and, although they rely on dedicated mining farms and hardware, their energy usage (estimated at 70 TWh/year in July 2021) is growing at a worrying pace [5]. Coupling these substantial carbon footprints with the growing global demand for computation warrants that we must both individually and collectively do our utmost to make our computations more environmentally friendly. Here, we describe 10 simple rules to help achieve this.

## Rule 1: Calculate the carbon footprint of your work

We live in a world ruled by data, where a problem doesn’t exist until it has been measured. There is still very limited information available about the carbon footprint of computational research, so the only way to appreciate the scale of the issue is for all of us to routinely calculate and report the carbon footprint of our work. This may seem intimidating at first, but, as we show below, estimating it can be quite straightforward. But before diving in, let’s pause for some useful concepts.

- **Carbon footprint:** This measures the environmental impact of an activity. Most of the time, carbon footprint measures the GHGs emitted during the activity, but it can also take into account the environmental impact of extracting the raw materials needed for example.
- **Carbon dioxide equivalent (CO**_**2**_**e):** Most activities are responsible for the emission of a variety of GHGs, not only carbon dioxide, each with different environmental impacts. For example, it is estimated that 1 kg of methane has the same impact on global warming as 21 kg of CO_2_ [6]. To help compare different activities, carbon footprints are presented in terms of “carbon dioxide equivalent” (CO_2_e), i.e., the quantity of CO_2_ having the same global warming impact as a given mix of GHGs.
- **The carbon footprint of everyday activities:** It is useful to put the carbon footprint of computation into context. For example, a European car emits on average 175 gCO_2_e/km [7,8] (251 gCO_2_e/km for an American car [9]), flying in Economy from Paris to London emits 50,000 gCO_2_e, and the footprint of flying from New York to San Francisco is almost 12 times higher (570,000 gCO_2_e) [10]. Streaming services also have a carbon footprint, and it has been recently estimated that streaming Netflix emits 55 gCO_2_e/hour [11]. Finally, trees are essential to eliminate excess CO_2_; on average, a mature tree can sequester 11,000 gCO_2_ per year [12].

The environmental impact of computing can be broadly divided between (i) the carbon footprint of powering the computers during the task itself; (ii) the impact of long-term data storage; and (iii) the life cycle footprint of the hardware.

Unsurprisingly, servers represent the largest share, over 50%, of a data centre’s energy usage [13]. Besides, when performing computation, servers’ power draw can be 4 times the idle power usage (i.e., when “sleeping”). This shows how crucial it is to estimate the carbon footprint of running an analysis. Since it depends on the energy needed to power the computer and the carbon footprint of producing such energy, it can be calculated fairly accurately. Specifically, by focusing on the elements with a significant impact on the carbon footprint, we can reduce the list of key parameters to geographic location, runtime, number and type of processors, memory available, and efficiency of the computing facility [14]. For example, the Green Algorithms calculator (www.green-algorithms.org) [14] can be used to estimate the carbon footprint of a task based on these parameters.

Hard drive storage accounts for about 10% of the electricity bill of a data centre [13]. Although it is significantly less than servers, it still represents around 20 TWh per year [2], similar to the total electricity generated by countries like Iceland or Tunisia [15]. The order of magnitude of the cradle-to-grave carbon footprint (i.e., including manufacturing, operation, and disposal) of hard drive storage is 10 kgCO_2_e per year and per TB of data [16]. Additionally, data centres often duplicate data on tapes transported by trucks to different locations. Put together, the impact of storage needs to be considered for projects relying on terabytes of data.

Finally, the end-to-end environmental impact of computers and data centres is substantial but difficult to quantify. In a life cycle assessment, all aspects are taken into account, from extraction of raw materials to transportation, manufacturing, and disposal [17]. One thing is certain, a clear way to limit your impact is to reduce technological waste.

## Rule 2: Include the carbon footprint in your cost–benefit analysis

When deciding whether to start a project, the estimated carbon footprint should be considered. This would both motivate researchers to reduce the environmental cost of their computation (using the Rules below) and shatter the illusion of “free compute time.” The concepts discussed in this article highlight important questions that need to be answered beforehand, such as “Is there a real need for speed if it comes at the expense of significant GHG emissions?,” “Can investing in new hardware be a sustainable solution?,” “Can we move our computation to a greener facility?,” and “Are funds available to offset our carbon footprint?.”

## Rule 3: Keep, repair, and reuse devices to minimise electronic waste

The devices that we use such as laptops, notepads, phones, etc., have significant environmental impacts. As of 2015, the global carbon footprint of manufacturing and using computers (laptop and desktop) was 150 Mt CO_2_e and 72 Mt CO_2_e for smartphones [18]. These devices have been heavily optimised to be energy efficient, and, in general, the energy consumption of our desktop setup is relatively low: between 70% and 80% of the cradle-to-grave carbon footprint of devices comes from the production life cycle [18]. For reference, the total carbon footprint of an iPhone 12 used for 3 years is 70 kgCO_2_e [19], and most recent smartphones are in the range 55 to 80 kgCO_2_e [18]. Unsurprisingly, laptops have a higher carbon footprint, around 280 kg CO_2_e: 185 kgCO_2_e for a 2020 13 inch MacBook Pro, but over twice as much (394 kgCO_2_e) for a 16 inch model (used 4 years) [19].

There are several ways to reduce the environmental impact of these devices. Firstly, do you need so many of them? Having fewer devices will reduce your total environmental impact. Secondly, try to use your gear for as long as is reasonable. As mentioned above, the carbon footprint of using a device is relatively small compared to the cost of producing it, and this usage footprint improves only marginally in new models (15 kgCO2e in an iPhone 4 from 2010 versus 13 kg in an iPhone X from 2017 [19]). This shows that limiting production footprint by keeping, maintaining, and passing on the same device for longer can drastically reduce our environmental impact. To achieve that, try to have your devices fixed rather than replaced. This requires manufacturers to make their products repairable (with detachable batteries for example) and although some companies like Fairphone work in this direction, easily fixable phones remain too rare. Finally, it’s important to ensure that devices are disposed of through the correct route. Informal recycling of electronic waste, which is generally labour intensive, low paid, and unregulated [20], causes high levels of air, soil, and water pollution in recycling areas (often in low- and middle-income countries), which poses serious risks to human health and the environment [21]. Remarkably, some devices like the Fairphone can even have a negative end-of-life footprint (i.e., a beneficial environmental impact) if recycled correctly, by recovering precious metal [22].

## Rule 4: Choose your computing facility

**Fig 1:**
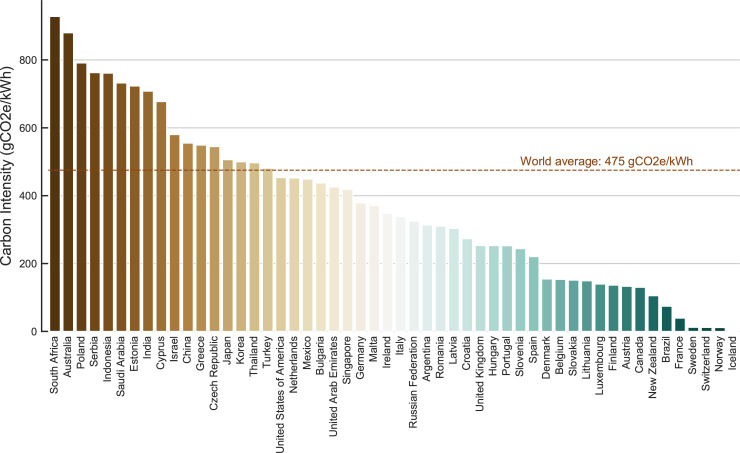
Carbon intensity (i.e., the carbon footprint of producing each kWh of electricity) for different countries, in gCO_2_e/kWh [23].

Due to the differences in energy production methods between countries, geographic location is perhaps the actionable factor with the greatest impact on carbon footprint (**Fig 1**). For example, the footprint of producing 1 kWh of electricity in Switzerland is 12 gCO_2_e, thanks to low-carbon energy production methods such as hydroelectricity and nuclear power. Australia, on the other hand, relies largely on coal and natural gas, which explains why producing the same kilowatt-hour has a carbon footprint of 880 gCO_2_e, 73 times larger [23].

Furthermore, regardless of the location, all computing facilities are not equal. Infrastructure overheads (mainly cooling) are responsible for 40% of the total electricity bill of an average data centre [24], but large optimised ones can reduce these overheads by 83%, so that they only represent 10% of the total electricity usage [25]. With cloud computing, you can both use optimised data centres and choose their location, reducing significantly energy needs and carbon footprint.

Finally, whether you use institutional data centres or cloud facilities, transparency regarding energy usage is crucial. Computational facilities need to be open about where the electricity they use comes from and their own energy efficiency.

## Rule 5: Choose your hardware carefully

Processors, memory, and runtime are the main parameters that control the energy usage, and the carbon footprint, of a task. By adjusting the type of processor used and the memory available, something that users can do on most high performance computing (HPC) platforms, the carbon footprint of a task can be greatly reduced. Among these, memory is perhaps the easiest one to act upon since, and this may come as a surprise, the energy needs depend almost entirely on the memory available, not on the memory actually used [26]. This means that allocating extra gigabytes to a task “just to be safe” or to avoid having to optimise the code (we have all done it) increases the carbon footprint substantially. The number and type of processors is often optimised to reduce runtime, through parallelisation or by replacing central processing units (CPUs) with graphics processing units (GPUs). However, the trade-off regarding carbon footprint can be complicated: Both these choices can sometimes increase the energy usage of a task despite reducing the total runtime. Whether these strategies can reduce both carbon footprint and runtime needs to be assessed for each situation.

Investing in more energy efficient hardware can be a way to reduce the carbon footprint of a task without compromising on performance. However, as we have discussed in Rule 3, it is important to take into account the environmental impact of producing the new equipment and disposing of the old one.

## Rule 6: Increase efficiency of the code

There are many avenues leading to more energy-efficient code, even if you don’t write the code yourself: One of the simplest ways to reduce the carbon footprint of your analysis is simply to ensure that you are using the most efficient software for the task. For example, we estimated that a biobank scale genome-wide association study (GWAS) with the v1 of BOLT-LMM emits a substantial 17 kgCO_2_e; however, simply updating to the latest v2.3 reduces the carbon footprint by 73% (to 5 kgCO_2_e) [27].

When writing code, keep in mind the key principles mentioned here: Limit peak memory requirements, be wary of hardware choices resulting in only small runtime improvements (which often come with large carbon footprints), and use recent optimised libraries when possible. In particular, code profilers such as RStudio’s built-in tool (and the “profvis” visualisation library) or the equivalent “cProfile” package in Python can be used to find sections that would benefit most from optimisation.

An algorithm’s carbon footprint is proportional to how many times it is used, and therefore, increasing the efficiency of a more popular tool will have a larger overall reduction in total GHG emissions. This is why, as a community, we propose that heavily utilised software should be prioritised for optimisation. Software developers should be aware of this proportionality as it is an avenue for them to limit the environmental impact of their work.

## Rule 7: Be a frugal analyst

As part of a project, an analysis is rarely performed just once. Often, the same computational pipeline is run several times to debug, optimise, and replicate, which multiplies the carbon footprint of the project. Keeping these duplications to a minimum is a painless way to reduce your carbon footprint. There are many ways to do that, we describe some below.

First, test and debug the entire pipeline on small example datasets; this reduces both the runtime and the memory requirements, which saves you time, money, and will significantly reduce your carbon footprint. Then, if despite careful debugging, some steps are known to have a high risk of failure, checkpoint your code (i.e., save intermediary states to resume from in case of error) to limit unnecessary computations.

Finding the optimal settings for a software requires extensive testing which causes disproportionate GHG emissions. Start by looking into the software’s documentation and public benchmarks to find optimal hardware choices and minimum memory requirements, which will be clearly identified if developers followed the next Rule. In some cases, it is possible to do the remaining tests on a smaller, representative dataset, and when it is not, strategies exist to reduce the number of trials and errors. For example, instead of testing all possible combinations of parameters, performing a random search has been shown to be more efficient and yields better results [28].

## Rule 8: Releasing a new software? Make its hardware requirements and carbon footprint clear

As discussed in Rule 5, selecting the type of hardware to use for an analysis—CPU or GPU, number of cores, and memory size—has a significant impact on the carbon footprint. To ensure that a new software is used as sustainably as possible, its developers should make it clear what the best hardware choices are for each situation.

In particular, it is crucial to detail how the memory requirements scale with the dimension of the input data. Otherwise, users tend to overallocate memory out of caution or underallocate memory and having to restart the analysis several times, both resulting in unnecessary GHG emissions. It is also useful to make it clear when increasing parallelisation or using GPUs instead of CPUs only results in marginal reductions in runtime at the expense of increased carbon footprint. Making this information available also enables future users to compare different tools, estimate the carbon footprint of a project ahead of the analysis, and include it in their cost–benefit analysis to make better informed choices; we talk more about this in Rule 2.

## Rule 9: Be aware of unanticipated consequences of improved software efficiency

We have witnessed incredible gains in speed of softwares over the last 2 decades, e.g., for DNA assembly and search. However, surprisingly, increasing the efficiency of a software doesn’t always lead to a reduction in compute. When given a faster implementation of an algorithm, a researcher will often imagine what larger questions might be addressed or run more analysis in the same timeframe. This rebound effect describes the situation where an increased efficiency in a process leads to greater increases in usage, in this case increasing the total carbon footprint. So you may need to make a conscious decision to keep efficiency wins without expanding the scope of the compute.

## Rule 10: Offset your carbon footprint

Carbon offsetting is complex, with debated benefits, and should never replace decreasing GHG emissions in the first place; however, it can mitigate the environmental impact of a project. Offsetting is flexible and can be implemented by a single user or by an institution.

There is a variety of offsetting initiatives investing in tree plantations, fuel efficient stoves, solar panels, or hydropower generators for example. Such initiatives, which tend to address both GHG emissions and other issues like food security, can be found on platforms such as Carbon Footprint [29]. Importantly, one should ensure that the projects are legitimate and certified by issuers that satisfy international standards set, for example, by the British Standard Institution. Examples of certified issuers are Gold Standard [30], Verra [31], and the American Carbon Registry [32].

In summary, if one wishes to invest in carbon offsetting, they should first ensure that they have minimised their carbon footprint as much as is feasible and then rely on certified offsetting projects.

## Conclusions

We have laid out our 10 simple rules that you can follow to make your computation more environmentally sustainable. We have discussed how to estimate and reduce one’s computational carbon footprint, the environmental impact of electronic hardware, and carbon offsetting. We have also detailed steps that computational researchers and software developers can follow and prioritise to reduce carbon footprints.

It is quite surprising that, given the data richness of computing science, it is still difficult to come up with accurate estimates of the environmental impact of computing. This is in part due to the diversity of computing platforms available, but also to the lack of reporting standards for hardware and software. However, in our experience, we can all get a sufficient understanding of our personal and institutional computational carbon footprint to make informed decisions and reduce our impact. We hope that our community invests time into creating new tools to understand the environmental impact of our work as well as promotes software (if not hardware) design practices that prioritise GHG minimisation.

Increasing the sustainability of computation is fundamental in tackling the current man-made climate emergency, and doing so requires everyone’s help. We believe that, with the rules displayed here, all of us can and must try to minimise our environmental impact.
